# Interleukin (IL)- 6 inhibition - Follow-up data of the German AID-registry^1^

**DOI:** 10.1186/1546-0096-13-S1-P63

**Published:** 2015-09-28

**Authors:** M Bielak, E Husmann, N Weyandt, JP Haas, G Horneff, T Lutz, E Lilienthal, T Kallinich, K Tenbrock, R Berendes, G Dückers, H Wittkowski, E Weißbarth-Riedel, G Heubner, PT Oommen, J Klotsche, U Neudorf, D Föll, T Niehues, E Lainka

**Affiliations:** 1 Universitätsklinikum Essen, Kinderklinik, Essen, Germany; 2 Klinik, Kinder- und Jugendrheumatologie, Garmisch-Partenkirchen, Germany; 3 Asklepios Klinik St. Augustin, Pädiatrie, St. Augustin, Germany; 4 Universitätsklinikum, Pädiatrie, Heidelberg, Germany; 5 Ruhr-Universität Bochum, Pädiatrie, Bochum, Germany; 6 Universitätsklinikum, Pädiatrie, Berlin, Germany; 7 Universitätsklinikum, Pädiatrie, Aachen, Germany; 8 Klinik, Pädiatrie, Landshut, Germany; 9 Helios Klinik, Pädiatrie, Krefeld, Germany; 10 Universitätsklinikum, Pädiatrische Rheumatologie, Münster, Germany; 11 Universitätsklinikum, Pädaitrische Rheumatologie, Hamburg, Germany; 12 Städtisches Krankenhaus, Pädiatrie, Dresden, Germany; 13 Universitätsklinikum, Pädiatrie, Düsseldorf, Germany; 14 DRFZ, Epidemiologie, Berlin, Germany

## Introduction

Systemic juvenile idiopathic arthritis (SJIA) is regarded as an autoinflammatory disease (AID) of unknown etiology related to abnormalities of the innate immune system. A major role in the pathogenesis has been ascribed to proinflammatory cytokines as interleukin (IL)-6 and IL-1.

## Objectives

Analysis of treatment results with the IL-6 inhibitor tocilizumab

## Patients and methods

From 7/2009 to 4/2014 200 patients with SJIA were documented in the AID-registry. 46 of 200 patients (19 m, 27 f) at the age of 1-18 years (median 9) received therapy with tocilizumab (median 13 months, range 1-48). 24 of 46 patients received long term treatment (median 23 months, range 12-48) and were evaluated concerning Wallace criteria [1]. Different clinical courses were continuous (C) n=12, polycyclic (PC) n=16, arthritic (A) n=18. Besides we estimated a response rate (definition: no clinical manifestation and no inflammation parameters) in the first 12 weeks of treatment. Data are based on the AID-Registry (http://www.aid-register.de).

## Results

According to Kaplan-Meier analysis 30% reached inactive disease or remission after the first 12 weeks of treatment. A rapid response to tocilizumab seems to be related to long term inactivity of SJIA. Comparison of the three disease courses (PC, C, A) revealed significant differences in the outcome; polycyclic courses show the fastest response followed by continuous courses. Worst outcome was evaluated in arthritic courses. Wallace criteria measured after at least 12 months: remission 54%, active disease 25%, inactive disease 21%. 4 (9%) patients were non-responders over the whole time. 60% of the patients showed no measurable CRP within the first 4 weeks and during tocilizumab therapy. Adverse events were reported in 11 (24%) patients: most leukopenia, infections and elevated transaminases, one Hodgkin's lymphoma, one gut perforation.

## Conclusion

A significant proportion of patients documented with SJIA in der German AID-registry is treated with tocilizumab (23%). We estimated a good response in the first 12 weeks of therapy of 30% and also by Wallace of 76% (inactive disease or remission). The response appears to depend on different disease phenotypes.

^1^The AID-Registry is funded by the BMBF (01GM08104, 01GM1112D)
